# Supplementary material for: Quantitative assessment of the nanoanatomy of the contractile vacuole complex in Trypanosoma cruzi
Source: Life Sci Alliance. 2024 Jul 29;7(10):e202402826. doi: 10.26508/lsa.202402826 (PMC11287019; doi:10.26508/lsa.202402826)
Supplement: Supplementary file 2 [file LSA-2024-02826_TableS1.docx]

**Table S1.** Volume of CVC in wild-type (WT), TcVps34 OE, and TcrPDEC2 OE mutants during systole stage of the CVC pulsation cycle.

|  | TcrPDEC2 OE | WT | TcVps34 OE |  |  |
| --- | --- | --- | --- | --- | --- |
| CV (nm³) | 5x10⁶ ± 1x10⁶ | 19.5x10⁶±6.5x10⁶ | 19x10⁶ ± 8.5x10⁶ |  |  |
| Spongiome (nm³) | 13,5x10⁶ ± 2x10⁶ | 11.5x10⁶±0.6x10⁶ | 30x10⁶ ± 4x10⁶** |  |  |
| Total (nm³) | 18,5x10⁶± 2.5x10⁶ | 31x10⁶± 6.5x10⁶ | 49x10⁶ ± 11x10⁶ |  |  |

Volume quantification is expressed as mean ± SEM. A one-way ANOVA test was applied. ** p = 0.002, n = 5.
