# Supplementary material for: Quantitative assessment of the nanoanatomy of the contractile vacuole complex in Trypanosoma cruzi
Source: Life Sci Alliance. 2024 Jul 29;7(10):e202402826. doi: 10.26508/lsa.202402826 (PMC11287019; doi:10.26508/lsa.202402826)
Supplement: Supplementary file 3 [file LSA-2024-02826_TableS2.docx]

**Table S2.** Volume of CVC in wild-type (WT), TcVps34 OE, and TcrPDEC2 OE cells during the diastole stage of the CVC pulsation cycle.

|  | TcrPDEC2 OE | WT | TcVps34 OE |  |  |
| --- | --- | --- | --- | --- | --- |
| CV (nm³) | 21.5x10⁶± 5x10⁶ | 32.5x10⁶±9.5x10⁶ | 54,5x10⁶±13,5x10⁶ |  |  |
| Spongiome (nm³) | 12.5x10⁶± 3x10⁶ | 8.5x10⁶± 3x10⁶ | 61.5x10⁶± 10x10⁶** |  |  |
| Total (nm³) | 34x10⁶ ± 3x10⁶ | 41x10⁶± 12x10⁶ | 116x10⁶± 25x10⁶* |  |  |

Volume quantification is expressed as mean ± SEM. A one-way ANOVA test was applied. * p ≤ 0.02, **p = 0.001, n = 5.
