# Supplementary material for: Quantitative assessment of the nanoanatomy of the contractile vacuole complex in Trypanosoma cruzi
Source: Life Sci Alliance. 2024 Jul 29;7(10):e202402826. doi: 10.26508/lsa.202402826 (PMC11287019; doi:10.26508/lsa.202402826)
Supplement: Supplementary file 6 [file LSA-2024-02826_TableS3.docx]

**Table S3.** Area of adhesion plaque in wild-type (WT), TcVps34 OE, and TcrPDEC2 OE cells at different stages of the CVC pulsation cycle.

|  | TcrPDEC2 OE | WT | TcVps34 OE |  |  |
| --- | --- | --- | --- | --- | --- |
| Systole | 4x10⁴ ± 1x10⁴ | 5x10⁴ ± 1.5x10⁴ | 7x10⁴ ± 1.5x10⁴ |  |  |
| Diastole | 8x10⁴ ± 2x10⁴ | 5x10⁴ ± 1.7x10⁴ | 14.5x10⁴ ± 1.2x10⁴ ** |  |  |

Values are expressed as the mean ± SEM in nm². A one-way ANOVA test was applied. ** p = 0.004, n = 5.
